# Surgical Rhizarthrosis Treatment: Trapezius Resection Arthroplasty Associated with Tendon Interposition versus the Kuhns Technique

**DOI:** 10.1055/s-0044-1788289

**Published:** 2024-09-04

**Authors:** Guilherme Henrique Teixeira Reis, Willker Galvão de Carvalho, Mauricio Foster Rodrigues, Jorge Raduan Neto, Aldo Okamura, João Carlos Belloti

**Affiliations:** 1 Departamento de Cirurgia da Mão, Hospital Alvorada, São Paulo, SP, Brasil

**Keywords:** orthopedic procedures, osteoarthritis, trapezium bone

## Abstract

**Objective** This study aimed to evaluate and compare the clinical and functional outcomes of two surgical procedures performed in patients with severe grade III and IV rhizarthrosis.

**Methods** We evaluated 39 patients who underwent two surgical techniques for rhizarthrosis treatment: trapeziectomy using the Kuhns technique or tendon interposition, with a minimum follow-up period of 6 months. The primary outcome assessment used the specific Trapeziometacarpal Arthrosis Symptoms and Disability (TASD) questionnaire, and the secondary outcome evaluation employed the shortened version of the Disabilities of the Arm, Shoulder, and Hand (QuickDASH) questionnaire and the visual analog scale (VAS).

**Results** There was no statistically significant difference between groups in the TASD, QuickDASH, and VAS results, and both techniques demonstrated good functional and pain outcomes. No complication required a new surgical approach. We found a positive correlation between TASD and QuickDASH questionnaire scores, suggesting their effectiveness in assessing functionality and disability in subjects with rhizarthrosis.

**Conclusion** Trapeziectomy using the Kuhns technique and tendon interposition proved effective in the surgical treatment of rhizarthrosis. There was no significant difference between the techniques concerning functional outcomes.

## Introduction

Rhizarthrosis is a progressive degenerative condition affecting the carpometacarpal joint of the thumb. It is one of the most common forms of osteoarthritis in the hands, and it is more frequent in women oldr than 50 years of age. This condition can be debilitating, limiting the subjects' daily living and work capacity. 1 Several therapeutic options are available to manage symptoms and improve quality of life, including lifestyle changes, motor and analgesic physical therapy, orthoses, pharmacological treatments, infiltrations, and surgery. 2

Surgical treatment is an option for patients with rhizarthrosis who do not obtain symptom relief through nonsurgical therapies. 2 Trapeziectomy is a resection arthroplasty of the trapezius that was first described is by Gervis, with good outcomes. 3 Some authors described the metacarpal's proximal migration and the compromised functional outcomes as complications of this technique. 4 Thus, to prevent these complications, techniques associated with trapeziectomy were described, including tendon interposition, 5 ligament reconstruction, 6 arthroplasty with implants, 7 the distraction hematoma formation technique (Kuhns technique), 8 ligament reconstruction with tendon interposition, 6 and acellular material interposition. 5 Although these associated techniques can be effective, they may increase the risk of other complications, such as infection, pain, implant loosening, and muscle strength loss. 4 The current literature has no studies with conclusive evidence regarding the most effective technique for the surgical treatment of rhizarthrosis. 4

Therefore, this study aimed to evaluate two surgical techniques: trapeziectomy with the Kuhns technique and with tendon interposition, in 39 patients, with a minimum follow-up period of 6 months.

These techniques were chosen due to the high number of patients who underwent these procedures to provide a comprehensive and representative analysis, allowing a significant comparison between them and contributing to validating the obtained results.

## Materials and Methods

This retrospective cohort study evaluated 39 patients. Inclusion criteria were the following: patients with clinical and imaging diagnosis of Eaton and Littler grade-III and -IV rhizarthrosis, 9 from both sexes, who underwent surgical treatment with trapeziectomy using the Kuhns technique 8 (group 1, n = 18), or trapeziectomy with tendon interposition 5 (group 2, n = 21), from 2018 to 2022, operated by four experienced hand surgeons, with a minimum follow-up period of 6 months.

Data were collected from the electronic records of the study's hospital, searching for patients with the following diagnosis codes of the International Classification of Diseases, Tenth Revision (ICD-10): M18.0, M18.1, and M19.9. Retrieved information included age, sex, operated side, dominant side, degree of osteoarthritis, and type of surgical procedure ( Table 1 ).

**Table 1:** Demographics of the patients

Data	Group 1	Group 2	Total	*p* -value
**Mean age (in years)**	62.4	58.0	60.1	0.056
**Sex**	94.5% F	90.5% F	92.4% F	0.052
	5.5% M	9.5% M	7.6% M	
**Dominant side**	94.5% R	81% R	87.2% R	0.921
	5.5% L	19% L	12.8% L	
**Operated side**	44.5% R	42.9% R	43.6% R	0.209
	55.5% L	57.1 L	56.4% L	
**Arthrosis grade** (Eaton and Littler)	67% III	72% III	70% III	
	33% IV	28% IV	30% IV	0.748
**Questionnaire application time (in months)**	Mean: 39.8	Mean: 21.6		
	Minimum: 8	Minimum: 6	Mean: 30	0,08
	Maximum: 62	Maximum: 50		

**Abbreviations:** III, grade III; IV, grade IV; F, female; L, left; M, male; R, right.

Exclusion criteria were the following: patients with rheumatological, traumatic, or neurological diseases affecting hand or wrist joints, those who underwent previous surgery in the thumb region, those lost to postoperative follow-up, and those who did not sign the informed consent form for the study.

After inclusion, we invited patients for an in-person assessment in a single outpatient visit with four residents in hand surgery. We asked the patients to answer questionnaires about their clinical and functional outcomes, including the Trapeziometacarpal Arthrosis Symptoms and Disability (TASD), 10 the shortened version of the Disabilities of the Arm, Shoulder, and Hand (QuickDASH), 11 and the Visual Analog scale (VAS) 12 for pain, as shown in the Annexes 1 , 2 , and 3 . The average time between the surgical procedure and the application of the questionnaires was 30 months.

For statistical analysis, we imported data to the IBM SPSS Statistics for MacOS (IBM Corp., Armonk, NY, USA) software, version 25.0. The descriptive statistics of categorical data included absolute and relative frequency. Continuous data underwent the Shapiro-Wilk normality test, and their description used mean ± standard deviation (SD), median, and 25th and 75th percentiles. Data with parametric distribution underwent the Student *t* test for two independent samples, while those with nonparametric distribution underwent the Mann-Whitney test for two independent samples. A difference was statistically significant when the type-I error, that is, the *p* -value, was lower than 0.05.

## Surgical Procedures Performed and Evaluated in the Study

**Fig. 1:**
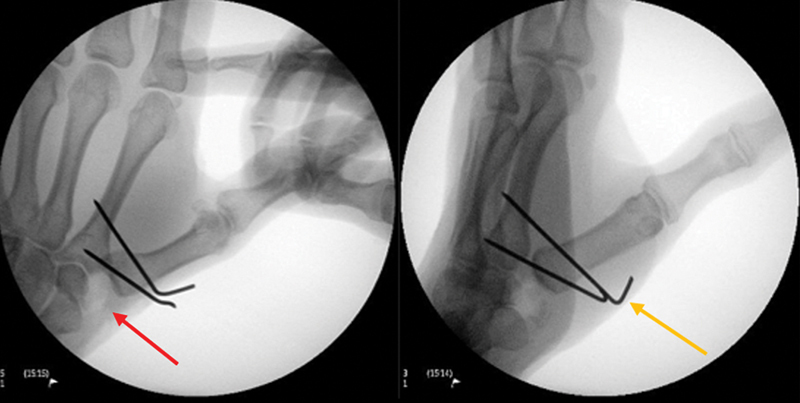
Completed surgical procedure using the Kuhns technique. Intraoperative images demonstrating the space (red arrow) after trapezium bone resection and the two Kirschner wires (yellow arrow) used to fixate the first to the second metacarpal bones.

For the first group, we performed the surgical technique described by Kuhns 8 using a longitudinal dorsal approach of approximately 4 cm in the carpometacarpal joint of the thumb between the tendon of the abductor pollicis longus muscle and the tendon of the extensor pollicis brevis muscle. Next, we opened the joint capsule in a T shape to expose and remove the trapezius; fixation was performed under radioscopy with two 1.5-mm Kirschner wires between the first and second metacarpals, maintaining the carpometacarpal joint space ( Fig. 1 ). The procedure ended with suturing the joint capsule and skin. Then, a sterile dressing and an antebracheopalmar plaster splint, including the thumb, were applied and kept for 4 weeks. We removed the Kirschner wires in the outpatient clinic 4 weeks after the surgery.

**Fig. 2:**
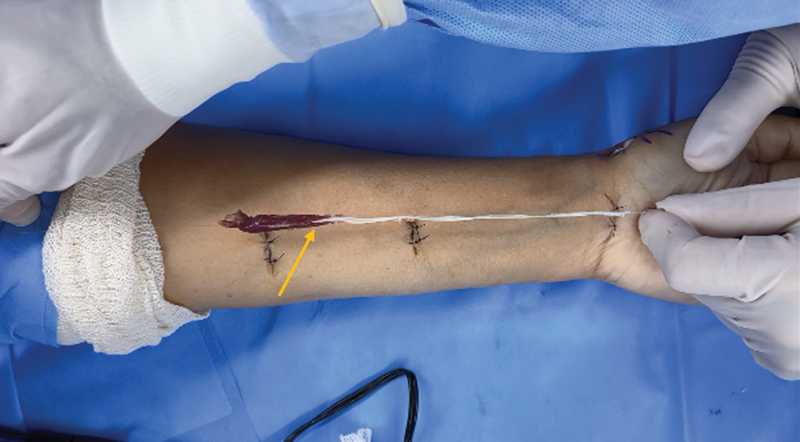
Image demonstrating the removal of the palmaris longus muscle for use as a graft. Palmaris longus muscle's musculotendinous graft (yellow arrow) removal from the three approaches.

**Fig. 3:**
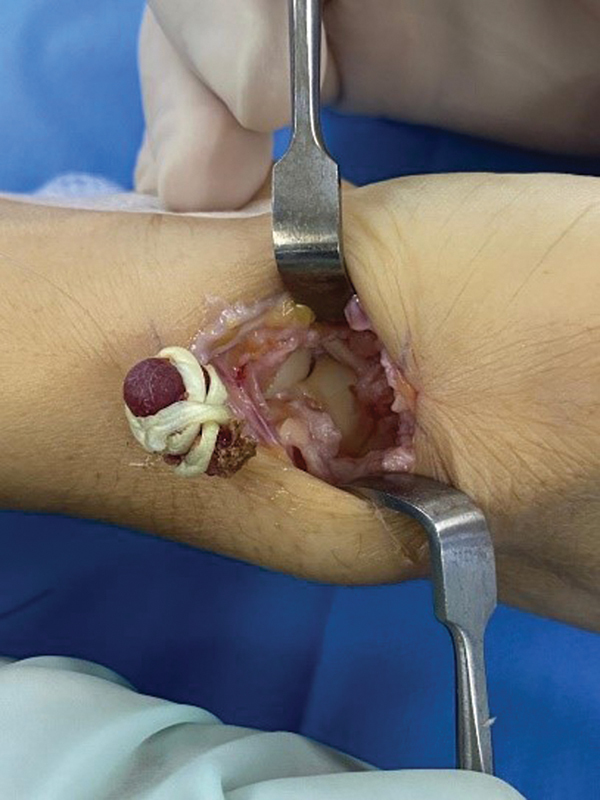
Palmaris longus muscle graft before being placed at the trapezius site.

As for the second group, a trapeziectomy and tenoarthroplasty technique was performed with the palmaris longus muscle, 5 using the same approach described for group 1. Then, we resected the tendon and the palmaris longus muscle using three approaches ( Fig. 2 ), creating a ball, and interposing it between the scaphoid and metacarpal bones ( Figs. 3 4 ). We closed the joint capsule ( Fig. 5 ), sutured the incision, and applied a bandage and a plaster splint. The sutures were removed after 2 weeks and immobilization was maintained for 4 weeks.

**Fig. 4:**
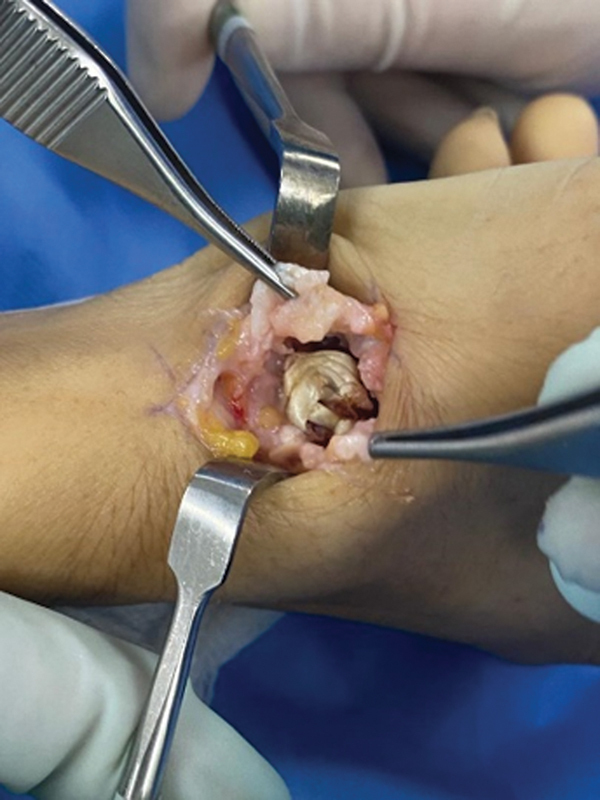
Graft placement at the site of the resected trapezius.

**Fig. 5:**
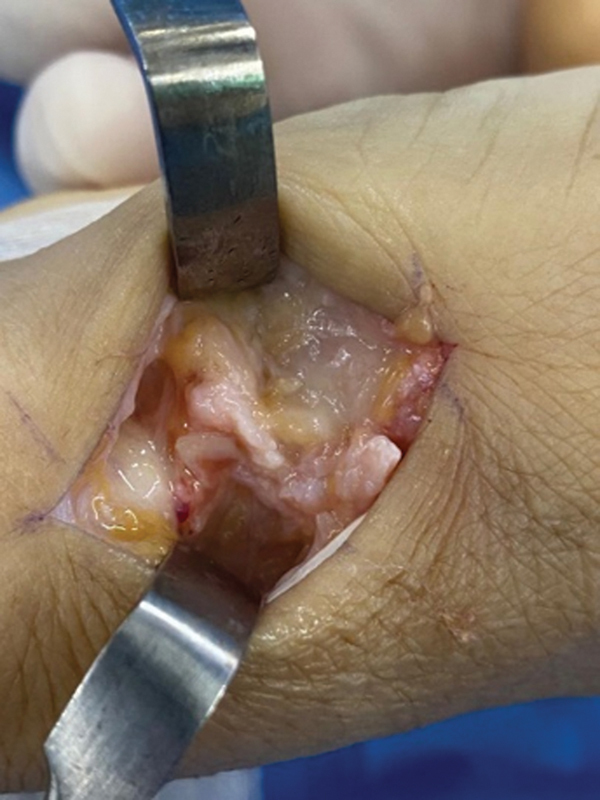
Joint capsule closure.

## Clinical Outcomes

### Primary Outcome

The primary outcome was the TASD questionnaire, 10 which consists of a specific self-reported assessment of rhizarthrosis-related functional limitations. The TASD was translated and culturally adapted to Brazilian Portuguese in 2021. 13 It contains a series of questions about pain intensity and functional thumb capacity. The answers are scored from 0 to 100, with higher scores indicating higher dysfunction.

### Secondary Outcomes

The QuickDASH 11 assesses upper limb functional disability and pain, listing 11 related activities. The answers are scored from 0 to 100, and higher scores indicate greater functional disability and pain reported by the patients.

The VAS 12 assesses the intensity of pain reported by patients. It consists of a horizontal line with a scale from 0 to 10, in which 0 represents no pain, and 10, maximum pain. Patients were asked to mark the degree of pain they were feeling at that moment on the scale.

A single evaluator not linked to the study applied the questionnaires during outpatient visits to ensure the standardization of data collection.

The ethics committee of our institution approved the study under the CAAE number 71550023.1.0000.5487.

## Results

The average time to apply the questionnaire to patients was of 30 months postoperatively, with a minimum time of 8 and a maximum of 62 months for group 1, and a minimum time of 6 and a maximum of 50 months for group 2 ( Table 1 ).

**Fig. 6:**
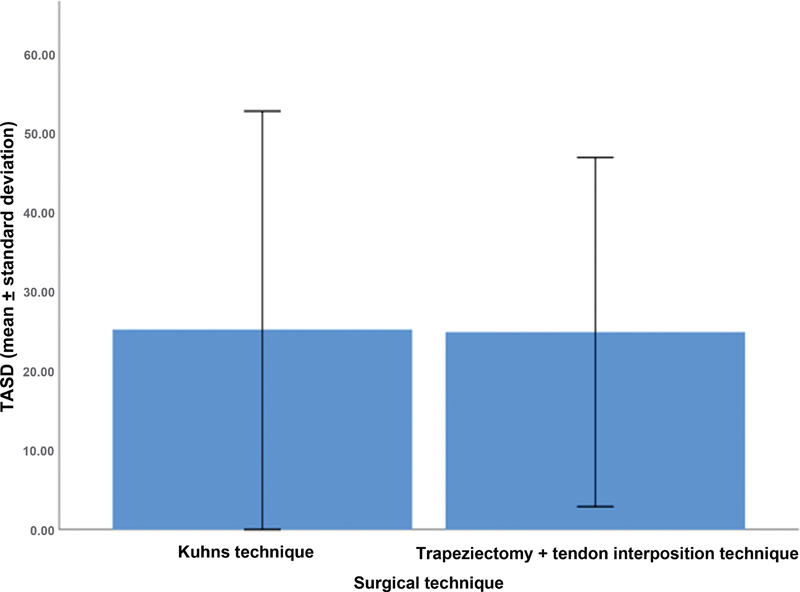
Analysis of the TASD scores in patients who underwent the Kuhns or tendon interposition techniques.

In the analysis of the primary outcome, the mean TASD score was of 25.2 ± 27.5% in the Kuhns technique group, and of 24.9 ± 22% in the tendon interposition group ( Fig. 6 ).

**Fig. 7:**
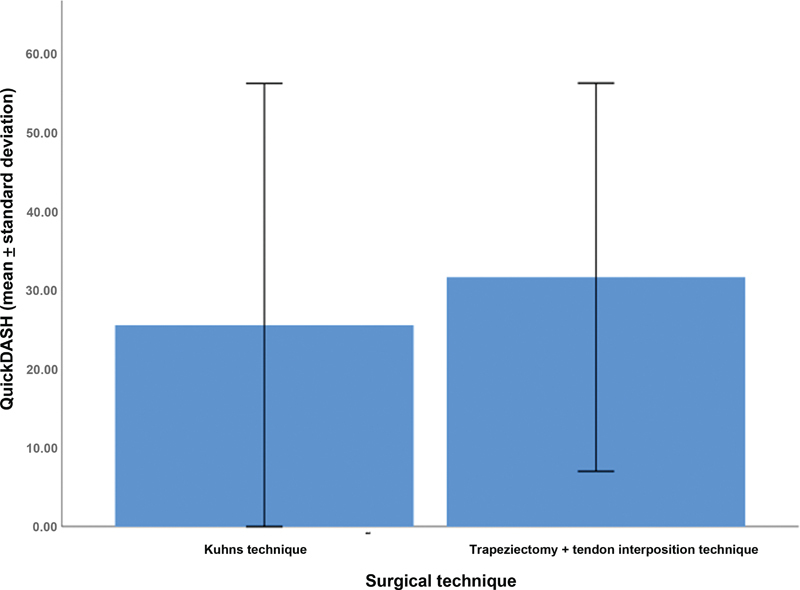
Analysis of the QuickDASH scores in patients who underwent the Kuhns or tendon interposition techniques.

In the analysis of the secondary outcomes, the mean QuickDASH score was of 25.5 ± 30.7% in the Kuhns technique group and of 31.6 ± 24.6% in the tendon interposition group ( Fig. 7 ).

**Fig. 8:**
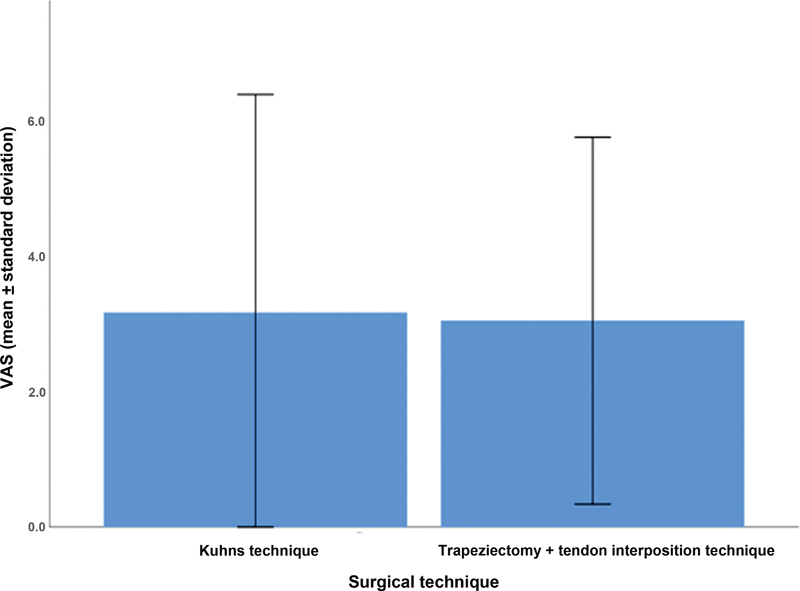
Analysis of the VAS scores in patients who underwent the Kuhns or trapeziectomy + tendon interposition techniques.

As for pain, the mean VAS score was of 3.2 ± 3.2% in the Kuhns technique group, and of 3.0 ± 2.7% in the tendon interposition group ( Fig. 8 ).

**Fig. 9:**
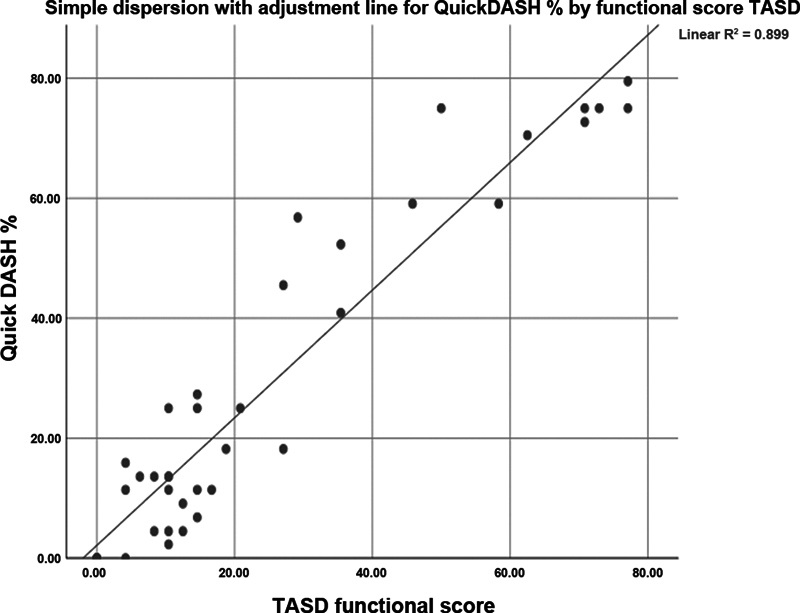
Correlation of the QuickDASH and TASD scores in postoperative patients who underwent the Kuhns or tendon interposition techniques.

We found a positive correlation between the TASD and QuickDASH scores: an increase in the score on one questionnaire corresponded to an increase in the score on the other questionnaire. This suggests that both tools are effective in measuring aspects related to functionality and disability in rhizarthrosis in a consistent manner ( Fig. 9 ).

## Discussion

The study sample was homogeneous and representative, as described in the literature. 1 This study used the TASD questionnaire as the primary outcome, and the QuickDASH questionnaire and the VAS as secondary outcomes, since these tools assess function and potential limitations in daily living activities and pain in operated patients. The literature reports that these are the most appropriate tools to evaluate the effectiveness of surgical treatment. 10 11

Our positive functional outcomes with the Kuhns technique 8 were consistent with those reported in the literature. 5 8 14 We observed as advantages of this technique, a shorter surgical time, the lack of need to make new incisions, and the exemption from tendon graft removal. These benefits simplify the procedure and result in a faster postoperative recovery, potentially reducing complications associated with additional incisions and grafting procedures. Its disadvantages are the potential complications inherent to the insertion and maintenance of the Kirschner wire and the need for a second procedure for pin removal, which occurred in the outpatient clinic of the institution in which the present study was conducted.

The technique of trapeziectomy with tenoarthroplasty of the palmaris longus muscle also seeks to avoid proximal migration of the first metacarpal bone by using the tendon graft as a biological spacer. The main advantage is the use of an autologous graft with no synthetic implant requirements. As the main disadvantages of this technique, we noted that, despite its performance on the same operated limb, it is limited for patients who lack the palmaris longus muscle and scarring complications from graft removal. The positive functional outcomes noted with the use of this technique in the present study are consistent with those of the literature, in which several authors 5 15 have reported good and excellent outcomes.

Comparing both surgical techniques, we observed that trapeziectomy with long palmar muscle tendon interposition was not superior to the Kuhns technique. Although we did not find studies specifically comparing both procedures, the outcomes were consistent with the literature addressing similar surgical techniques. 16 17 18 19 The findings of this review support the effectiveness of both approaches in rhizarthrosis treatment. However, based on the Cochrane Review, 20 we cannot currently make recommendations regarding the superiority of any surgical procedure over another for this condition.

We observed a positive correlation between the TASD a QuickDASH, a result consistent with the findings in the literature. 10 21

## Conclusion

The two techniques evaluated proved effective for treating patients with rhizarthrosis per the TASD, with an average postoperative follow-up of 30 months. There was no superiority in functional outcomes between the groups when comparing trapeziectomy techniques with tendon interposition or distraction. The specific TASD and the generic QuickDASH functional questionnaires proved equivalent to measuring the patients' degree of functional limitation.
