# Supplementary material for: Surgical Rhizarthrosis Treatment: Trapezius Resection Arthroplasty Associated with Tendon Interposition versus the Kuhns Technique
Source: Rev Bras Ortop (Sao Paulo). 2024 Sep 4;59(4):e572–9. doi: 10.1055/s-0044-1788289 (PMC11374393; doi:10.1055/s-0044-1788289)
Supplement: Supplementary file 1 — Supplementary Material [file 10-1055-s-0044-1788289_s2300288pt.pdf]

*Quick*DASH

Por favor meça sua habilidade para realizar as seguintes atividades na semana passada circulando o número apropriado da resposta:

	Não houve dificuldade	Houve pouca dificuldade	Houve dificuldade moderada	Dificuldade severa	Não consegui u fazer
1. Abrir um vidro novo ou com a tampa muito apertada.	1	2	3	4	5
2. Fazer tarefas domésticas pesadas (por exemplo: lavar paredes, lavar o chão).	1	2	3	4	5
3. Carregar uma sacola ou uma maleta.	1	2	3	4	5
4. Lavar suas costas.	1	2	3	4	5
5. Usar uma faca para cortar alimentos.	1	2	3	4	5
6. Atividades recreativas que exigem alguma força ou impacto nos braços, ombros ou mãos (por exemplo: jogar vôlei, martelar).	1	2	3	4	5

	Não afetou	Afetou pouco	Afetou Moderadamen te	Afetou muito	Afetou Extrema Mente
7. Durante a semana passada, em que ponto o seu problema com braço, ombro ou mão afetaram suas atividades normais com família, amigos, vizinhos ou colegas?	1	2	3	4	5

	Não limitou	Limitou pouco	Limitou moderadamen te	Limitou muito	Não conseguiu fazer
8. Durante a semana passada, o seu trabalho ou outras atividades diárias regulares foram limitadas devido ao seu problema com braço, ombro ou mão?	1	2	3	4	5

Por favor meça a gravidade dos seguintes sintomas na semana passada. (circule o número)	Nenhuma	Pouca	Moderada	Severa	Extrema
9. Dor no braço, ombro ou mão.	1	2	3	4	5
10. Desconforto na pele (alfinetadas) no braço, ombro ou mão.	1	2	3	4	5

	Não houve dificuldade	Pouca dificuldade	Dificuldade moderada	Dificuldade severa	Tão difícil que eu não pude dormir
11. Durante a semana passada, quanto de dificuldade você teve para dormir por causa da dor no seu braço, ombro ou mão? (circule o número)	1	2	3	4	5

Anexo 1 Questionário QuickDASH.

 © 2024. The Author(s).

Assinale o número correspondente a dor que você sente no seu polegar. *

	0	1	2	3	4	5	6	7	8	9	10	
Nenhuma dor	○	○	○	○	○	○	○	○	○	○	○	Máximo de dor

Anexo 2 EVA para dor.

TASD-BR: Responda o Questionário com apenas uma alternativa:

Por favor, diga a intensidade dos seguintes sintomas:

1. Você sente dor na base dos seu polegar (próximo ao punho), sem atividades (mesmo quando não mexe o polegar)?
   1. NENHUMA
   2. LEVE
   3. MODERADA
   4. INTENSA
   5. MUITO INTENSA

2. Você sente dor na base dos seu polegar (próximo ao punho) durante atividades (quando mexe o polegar)?
   1. NENHUMA
   2. LEVE
   3. MODERADA
   4. INTENSA
   5. MUITO INTENSA

3. Você sente desconforto (incômodo) na base do polegar (próximo ao punho) quando aperta ou encosta em alguma coisa?
   1. NENHUM
   2. LEVE
   3. MODERADO
   4. INTENSO
   5. MUITO INTENSO

4. Você percebe inchaço (edema) na base do polegar (próximo ao punho)?
   1. NENHUM
   2. LEVE
   3. MODERADO
   4. INTENSO
   5. MUITO INTENSO

5. Você percebe Rigidez (endurecimento) na base do polegar (próximo ao punho) quando faz algum movimento?
   1. NENHUM (A)
   2. LEVE
   3. MODERADO (A)
   4. INTENSO (A)
   5. MUITO INTENSO (A)

6. Quanto você perdeu de movimento do polegar?
   1. NENHUM
   2. LEVE
   3. MODERADO
   4. INTENSO
   5. MUITO INTENSO

7. Você sente diminuição de força quando pega e segura algum objeto?
   1. NENHUMA
   2. LEVE
   3. MODERADA
   4. INTENSA
   5. MUITO INTENSA

Por favor avalie quanta dificuldade você tem ao fazer as seguintes atividades:

1. Abrir um pote ou garrafa com tampa de rosca apertada ou nova
   1. SEM DIFICULDADE
   2. UM POUCO DE DIFICULDADE
   3. DIFICULDADE MODERADA
   4. MUITA DIFICULDADE
   5. NÃO CONSEGUE FAZER

2. Virar uma chave (de uma porta)
   1. SEM DIFICULDADE
   2. UM POUCO DE DIFICULDADE
   3. DIFICULDADE MODERADA
   4. MUITA DIFICULDADE
   5. NÃO CONSEGUE FAZER

3. Girar a maçaneta redonda (do tipo bola) da porta
   1. SEM DIFICULDADE
   2. UM POUCO DE DIFICULDADE
   3. DIFICULDADE MODERADA
   4. MUITA DIFICULDAE
   5. NÃO CONSEGUE FAZER

4. Fechar o zíper (fecho-ecler)
   1. SEM DIFICULDADE
   2. UM POUCO DE DIFICULDADE
   3. DIFICULDADE MODERADA
   4. MUITA DIFICULDAE
   5. NÃO CONSEGUE FAZER

5. Segurar objetos grandes (copo, garrafa, livro, etc.)
   1. SEM DIFICULDADE
   2. UM POUCO DE DIFICULDADE
   3. DIFICULDADE MODERADA
   4. MUITA DIFICULDAE
   5. NÃO CONSEGUE FAZER

Anexo 3 Questionário TASD-BR.

 © 2024. The Author(s).
