# Supplementary material for: Surgical Rhizarthrosis Treatment: Trapezius Resection Arthroplasty Associated with Tendon Interposition versus the Kuhns Technique
Source: Rev Bras Ortop (Sao Paulo). 2024 Sep 4;59(4):e572–9. doi: 10.1055/s-0044-1788289 (PMC11374393; doi:10.1055/s-0044-1788289)
Supplement: Supplementary file 2 — Supplementary Material [file 10-1055-s-0044-1788289_s2300288en.pdf]

*Quick*DASH

Please rate your ability to do the following activities in the last week by circling the number below the appropriate response.

	No difficulty	A little difficulty	Moderate difficulty	Severe difficulty	Unable
1. Open a tight or new jar	1	2	3	4	5
2. Do heavy household chores (e.g., wash walls, floors).	1	2	3	4	5
3. Carry a shopping bag or briefcase.	1	2	3	4	5
4. Wash your back.	1	2	3	4	5
5. Use a knife to cut food.	1	2	3	4	5
6. Recreational activities in which you take some force or impact through your arm, shoulder, or hand (e.g., golf, hammering, tennis, etc.).	1	2	3	4	5

	Not at all	Slightly	Moderately	Quite a bit	Extremely
7. During the past week, to what extent has your arm, shoulder, or hand problem interfered with your normal social activities with family, friends, neighbors or colleagues?	1	2	3	4	5

	Not limited at all	Slightly limited	Moderately limited	Very limited	Unable
8. During the past week, were you limited in your work or other regular daily activities as a result of your arm, shoulder, or hand problem?	1	2	3	4	5

Please rate the severity of the following symptoms in the last week. (circle number)	None	Mild	Moderate	Severe	Extreme
9. Arm, shoulder, or hand pain.	1	2	3	4	5
10. Tingling (pins and needles) in your arm, shoulder, or hand.	1	2	3	4	5

	No difficulty	A little difficulty	Moderate difficulty	Severe difficulty	So difficult that I can't sleep
11. During the past week, how much difficulty have you had sleeping because of the pain in your arm, shoulder, or hand? (circle number)	1	2	3	4	5

Annex 1 QuickDASH questionnaire.

 © 2024. The Author(s).

Mark the number corresponding to the pain you feel in your thumb. *

	0	1	2	3	4	5	6	7	8	9	10	
No pain	○	○	○	○	○	○	○	○	○	○	○	Maximum pain

Annex 2 VAS for pain.

TASD – Answer the Questionnaire with a single alternative:

Please rate the following symptoms:

1. Do you feel pain at the base of your thumb (next to the wrist) at rest (even when not moving your thumb)?
   1. NONE
   2. MILD
   3. MODERATE
   4. SEVERE
   5. EXTREME

2. Do you feel pain at the base of your thumb (next to the wrist) during activities (when moving your thumb)?
   1. NONE
   2. MILD
   3. MODERATE
   4. SEVERE
   5. EXTREME

3. Do you feel tenderness at the base of your thumb (next to the wrist) when grasping or touching something?
   1. NONE
   2. MILD
   3. MODERATE
   4. SEVERE
   5. EXTREME

4. Is there swelling at your thumb (next to your wrist)?
   1. NONE
   2. MILD
   3. MODERATE
   4. SEVERE
   5. EXTREME

5. Is there rigidity (stiffness) at the base of your thumb (next to the wrist) when you perform some movement?
   1. NONE
   2. MILD
   3. MODERATE
   4. SEVERE
   5. EXTREME

6. How much range of motion have you lost in your thumb?
   1. NONE
   2. MILD
   3. MODERATE
   4. SEVERE
   5. EXTREME

7. Do you feel decreased strength when pinching or grasping objects?
   1. NONE
   2. MILD
   3. MODERATE
   4. SEVERE
   5. EXTREME

Please rate how much difficulty you have had doing the following activities:

1. Open a new or tight jar
   1. NO DIFFICULTY
   2. A LITTLE DIFFICULTY
   3. MODERATE DIFFICULTY
   4. SEVERE DIFFICULTY
   5. UNABLE

2. Turn a key
   1. NO DIFFICULTY
   2. A LITTLE DIFFICULTY
   3. MODERATE DIFFICULTY
   4. SEVERE DIFFICULTY
   5. UNABLE

3. Turn a doorknob
   1. NO DIFFICULTY
   2. A LITTLE DIFFICULTY
   3. MODERATE DIFFICULTY
   4. SEVERE DIFFICULTY
   5. UNABLE

4. Pull a zipper
   1. NO DIFFICULTY
   2. A LITTLE DIFFICULTY
   3. MODERATE DIFFICULTY
   4. SEVERE DIFFICULTY
   5. UNABLE

5. Grasp larger objects (glass, bottle, book, etc.)
   1. NO DIFFICULTY
   2. A LITTLE DIFFICULTY
   3. MODERATE DIFFICULTY
   4. SEVERE DIFFICULTY
   5. UNABLE

Annex 3 TASD-BR questionnaire.

 © 2024. The Author(s).
