# Supplementary material for: Surgical Rhizarthrosis Treatment: Trapezius Resection Arthroplasty Associated with Tendon Interposition versus the Kuhns Technique
Source: Rev Bras Ortop (Sao Paulo). 2024 Sep 4;59(4):e572–9. doi: 10.1055/s-0044-1788289 (PMC11374393; doi:10.1055/s-0044-1788289)
Supplement: Related texts

# Research article: Tratamento cirúrgico da rizartrose: Artroplastia de ressecção do trapézio associada a interposição tendínea *versus* técnica de Kuhns

**Keywords:** osso trapézio, osteoartrite, procedimentos ortopédicos

## Abstract

**Objetivo** O objetivo deste estudo é avaliar e comparar os resultados clínicos e funcionais de dois procedimentos cirúrgicos realizados em pacientes com rizartrose grave graus III e IV.

**Métodos** Avaliamos 39 pacientes submetidos a 2 técnicas cirúrgicas para o tratamento da rizartrose: trapeziectomia pela técnica de Kuhns ou com interposição tendínea, com mínimo de 6 meses de seguimento. O desfecho primário foi avaliado pelo questionário específico Sintomas e Incapacidade da Artrose Trapeziometacarpiana (Trapeziometacarpal Arthrosis Symptoms and Disability, TASD, em inglês), e os desfechos secundários, pela versão abreviada do Questionário de Incapacidades do Braço, Ombro e Mão (Disabilities of the Arm, Shoulder, and Hand, QuickDASH, em inglês) e a Escala Visual Analógica (EVA).

**Resultados** Não houve diferença estatisticamente significantiva entre os grupos nos resultados do TASD, QuickDASH e EVA e ambas as técnicas demonstraram bons resultados funcionais e da dor. Não houve complicações que necessitassem de nova abordagem cirúrgica. Verificamos uma correlação positiva entre as pontuações no TASD e no QuickDASH, o que sugere que ambos são eficazes na avaliação da funcionalidade e incapacidade da rizartrose.

**Conclusão** A trapeziectomia com técnica de Kuhns e com interposição tendínea se mostraram efetivas no tratamento cirúrgico da rizartrose. Não houve diferença significativa entre as técnicas em relação aos resultados funcionais.

## Introdução

A rizartrose, é uma condição degenerativa progressiva que afeta a articulação carpometacarpiana do polegar. É uma das formas mais comuns de osteoartrite nas mãos, sendo mais frequente em mulheres acima dos 50 anos de idade. Essa condição pode ser debilitante, limitando a capacidade de vida e laboral diária do indivíduo. 1 Várias opções terapêuticas estão disponíveis para gerenciar os sintomas e melhorar a qualidade de vida, como as mudanças no estilo de vida, fisioterapia motora e analgésica, órteses, terapias farmacológicas, infiltrações e tratamentos cirúrgicos. 2

O tratamento cirúrgico é uma opção para pacientes com rizartrose que não obtiveram alívio dos sintomas por meio de tratamentos não cirúrgicos. 2 A trapeziectomia é uma artroplastia de ressecção do trapézio que foi inicialmente descrita por Gervis, com bons resultados. 3 Alguns autores descreveram como complicação desta técnica a migração proximal do metacarpo e o comprometimento dos resultados funcionais. 4 Assim, para prevenir tais complicações, foram descritas técnicas associadas a trapeziectomia como a interposição de tendão, 5 a reconstrução ligamentar, 6 artroplastia com implantes, 7 a técnica de formação de hematoma por distração (técnica de Kuhns), 8 reconstrução ligamentar com a interposição de tendão, 6 e interposição de material acelular. 5 Embora estas técnicas associadas possam ser efetivas, elas podem acrescentar riscos de outras complicações como infecção, dor, soltura dos implantes e perda de força muscular. 4 Atualmente na literatura não há estudos com evidências conclusivas quanto a técnica mais efetiva para o tratamento cirúrgico da rizartrose. 4

Assim, o objetivo deste estudo é avaliar duas técnicas cirúrgicas: a trapeziectomia com a técnica de Kuhns e com interposição tendinosa, em 39 pacientes, com um segmento mínimo de 6 meses de evolução.

Essas técnicas foram escolhidas devido ao considerável número de pacientes submetidos a esses procedimentos com o intuito de proporcionar uma análise abrangente e representativa, permitindo uma comparação significativa entre elas e contribuindo para a validade dos resultados obtidos.

## Materiais e Métodos

Este é um estudo coorte retrospectivo que avaliou 39 pacientes. Os critérios de inclusão foram: pacientes com diagnóstico clinico e de imagem com rizartrose de graus III e IV de Eaton e Littler, 9 de ambos os sexos, que foram submetidos ao tratamento cirúrgico com a trapeziectomia pela técnica de Kuhns 8 (grupo 1, n = 18), ou a trapeziectomia associada com interposição de tendão 5 (grupo 2, n = 21), entre os anos de 2018 e 2022, operados por quatro cirurgiões de mão experientes e com seguimento mínimo de seis meses.

Os dados foram coletados a partir dos registros eletrônicos do hospital sede do estudo, com busca dos pacientes por meio dos códigos de diagnóstico da décima revisão da Classificação Internacional de Doenças (CID-10): M18.0, M18.1 e M19.9. As informações coletadas incluíram idade, sexo, lado operado, lado dominante, grau de artrose e tipo de procedimento cirúrgico ( Tabela 1 ).

**Tabela 1:** Dados demográficos dos pacientes

Dados	Grupo 1	Grupo 2	Total	Valor de *p*
**Idade média (em anos)**	62,4	58,0	60,1	0,056
**Sexo**	94,5% F	90,5% F	92,4% F	0,052
	5,5% M	9,5% M	7,6% M	
**Lado dominante**	94,5% D	81% D	87,2% D	0,921
	5,5% E	19% E	12,8% E	
**Lado operado**	44,5% D	42,9% D	43,6% D	0,209
	55,5% E	57,1 E	56,4% E	
**Grau de artrose** (Eaton e Littler)	67% III	72% III	70% III	
	33% IV	28% IV	30% IV	0,748
**Tempo da aplicação do questionário (em meses)**	Média: 39,8	Média: 21,6		
	Mínimo: 8	Mínimo: 6	Média: 30	0,08
	Máximo: 62	Máximo: 50		

**Abreviaturas:** III, grau III; IV, grau IV; D, direito; E, esquerdo; F, feminino; M, masculino.

Os critérios de exclusão foram: pacientes com doenças reumatológicas, traumáticas, ou neurológicas que afetam as articulações da mão ou do punho, aqueles submetidos a cirurgias prévias na região do polegar, aqueles que perderam o seguimento pós-operatório e aqueles que não assinaram o termo de consentimento do estudo.

Após inclusão, os pacientes foram convidados a realizar uma avaliação presencial em consultas ambulatoriais, em um único momento, por quatro residentes em cirurgia da mão. Foi solicitado aos pacientes que respondessem aos questionários sobre os seus desfechos clínicos e funcionais, sendo eles o questionário de Sintomas e Incapacidade da Artrose Trapeziometacarpiana (Trapeziometacarpal Arthrosis Symptoms and Disability, TASD, em inglês), 10 a versão abreviada do Questionário de Incapacidades do Braço, Ombro e Mão (Disabilities of the Arm, Shoulder, and Hand, QuickDASH, em inglês) 11 e a Escala Visual Analógica (EVA) de dor, 12 como demonstrado nos Anexos 1 , 2 e 3 . O tempo médio entre o procedimento cirúrgico e a aplicação dos questionários foi de 30 meses.

Na análise estatística, os dados foram importados para o programa IBM SPSS Statistics for MacOS (IBM Corp., Armonk, NY, EUA), versão 25.0. A estatística descritiva de dados categóricos foi descrita pela sua frequência absoluta e relativa. Os dados contínuos foram submetidos ao teste de normalidade de Shapiro-Wilk e descritos pela média ± desvio padrão, mediana e pelos 25 ^o^ e 75 ^o^ percentis. Os dados que mostraram distribuição paramétrica foram submetidos ao teste *t* de Student para duas amostras independentes, enquanto os dados que não mostraram distribuição paramétrica foram submetidos ao teste Mann-Whitney para duas amostras independentes. Foi aceito como diferença estatisticamente significativo quando o erro do tipo I, ou seja, o valor de *p* , foi menor do que 0,05.

## Procedimentos Cirúrgicos Realizados e Avaliados no Estudo

Para o primeiro grupo, foi realizada a técnica cirúrgica descrita por Kuhns, 8 mediante via acesso dorsal longitudinal de cerca de 4 cm, na articulação carpometacarpiana do polegar entre o tendão do musculo abdutor longo do polegar e o tendão do musculo extensor curto do polegar. Em seguida, a capsula articular foi aberta em T para expor e remover o trapézio; a fixação foi realizada sob radioscopia com dois fios de Kirschner 1.5mm entre o primeiro e o segundo metacarpo, mantendo o espaço articular carpometacarpiano ( Fig. 1 ). O procedimento é finalizado com a sutura da capsula articular e da pele. Então, é feito um curativo estéril e tala gessada antebraqueopalmar com inclusão do polegar, que é mantida por 4 semanas. Os fios de Kirschner foram retirados no ambulatório com 4 semanas de pós-operatório.

**Fig. 1:** Procedimento cirúrgico finalizado pela técnica de Kuhns. Imagens do intraoperatório que demonstram o espaço (seta vermelha) após a ressecção do osso trapézio e dos dois fios de Kirschner (seta amarela) utilizados para fixar o primeiro metacarpo ao segundo metacarpo.

Quanto ao segundo grupo, a técnica de trapeziectomia e tenoartroplastia com o músculo palmar longo, 5 foi realizada pela mesma via de acesso descrita no grupo 1, a ressecção do tendão e do musculo palmar longo é realizada com o uso de três acessos sobre o tendão ( Fig. 2 ), a confecção do novelo e a sua interposição é realizada entre o escafoide e o metacarpo ( Figs. 3 4 ). A capsula articular ( Fig. 5 ) é fechada seguida da sutura da incisão e um curativo e tala gessada foram aplicados, os pontos são retirados com 2 semanas e a imobilização é mantida por 4 semanas.

**Fig. 2:** Imagem da retirada do músculo palmar longo para ser utilizado como enxerto. Retirada do enxerto musculotendíneo do músculo palmar longo (seta amarela) por meio de três acessos.

**Fig. 3:** Enxerto do musculo palmar longo antes de ser colocado no local do trapézio.

**Fig. 4:** Colocação do enxerto no local do trapézio ressecado.

**Fig. 5:** Fechamento da capsula articular.

## Desfechos Clínicos

## Desfecho Primário

O desfecho primário foi utilizado o questionário específico TASD, 10 que consiste em uma avaliação autorreportada específica das limitações funcionais relacionadas à rizartrose. O TASD foi traduzido e adaptado culturalmente para o português brasileiro em 2021. 13 Ele contém uma série de perguntas sobre a intensidade da dor e a capacidade funcional para o polegar. As respostas são pontuadas em uma escala de 0 a 100, e pontuações mais altas indicam maior disfunção.

## Desfechos Secundários

O QuickDASH 11 avalia a incapacidade funcional e a dor relacionadas ao membro superior por meio da listagem de 11 atividades. As respostas são pontuadas em uma escala de 0 a 100 e, quanto mais alta a pontuação, maior a incapacidade funcional e a dor relatadas pelos pacientes.

A EVA 12 avalia a intensidade da dor relatada pelos pacientes. Consiste em uma linha horizontal com uma escala de 0 a 10, em que 0 representa ausência de dor e 10, dor máxima. Os pacientes foram convidados a marcar na escala o grau de dor que estavam sentindo naquele momento.

Os questionários foram aplicados por um único avaliador não ligado ao estudo, em todas as consultas ambulatoriais, garantindo a padronização da coleta de dados.

O trabalho foi aprovado pelo comitê de ética da nossa instituição sob o número CAAE: 71550023.1.0000.5487.

## Resultados

O tempo médio para a aplicação dos questionários nos pacientes foi de 30 meses pós-operatórios, sendo para o grupo 1 um tempo mínimo de 8 e máximo de 62 meses, e para o grupo 2 um tempo mínimo de 6 e máximo de 50 meses ( Tabela 1 ).

Na análise do desfecho primário pela pontuação no TASD, o grupo da técnica de Kuhns teve uma média de 25,2 ± 27,5%, ao passo que, no grupo de interposição de tendão, a média foi de 24,9 ± 22% ( Fig. 6 ).

**Fig. 6:** Análise da pontuação no TASD em comparação com as técnicas de Kuhns e de interposição de tendão.

Na análise do desfechos secundários pela pontuação no QuickDASH, o grupo da técnica de Kuhns teve média de 25,5 ± 30,7%, e no grupo da interposição de tendão, a média foi de 31,6 ± 24,6% ( Fig. 7 ).

**Fig. 7:** Análise da pontuação no QuickDASH em comparação com as técnicas de Kuhns e de interposição de tendão.

Na análise do desfecho de dor, aferido pela EVA, o grupo da técnica de Kuhns, teve média de 3,2 ± 3,2%, e no grupo da interposição de tendão, a média foi de 3,0 ± 2,7% ( Fig. 8 ).

**Fig. 8:** Análise da pontuação na EVA em comparação com as técnicas de Kuhns e de interposição de tendão.

Foi encontrada uma correlação positiva entre as pontuações no TASD e QuickDASH: um aumento nas pontuações em um questionário correspondia a um aumento nas pontuações no outro. Isso sugere que ambos os instrumentos são eficazes para medir aspectos relacionados à funcionalidade e incapacidade na rizartrose de forma consistente ( Fig. 9 ).

**Fig. 9:** Correlação das pontuações no QuickDASH e TASD nos pacientes pós-operatórios que se submeteram às técnicas de Kuhns e de interposição de tendão.

## Discussão

A amostra populacional do estudo se mostrou homogênea e representativa, conforme descrito na literatura. 1 Este estudo utilizou como desfecho primário o questionário TASD, e como secundários o questionário QuickDASH e a EVA, por serem instrumentos que avaliam a função e possíveis limitações de atividades da vida diária e o índice de dor dos pacientes operados. A literatura relata que essas são as ferramentas mais apropriadas para avaliar a efetividade do tratamento cirúrgico. 10 11

Os nossos resultados funcionais positivos com a técnica descrita por Kuhns 8 foram concordantes com os da literatura. 5 8 14 Observamos como vantagens dessa técnica um menor tempo cirúrgico, a falta de necessidade de realizar novas incisões e a dispensa da retirada de enxertos tendíneos. Esses benefícios não apenas simplificam o procedimento, como também podem resultar em uma recuperação pós-operatória mais rápida, o que pode reduzir as complicações associadas a incisões adicionais e procedimentos de enxertos. Suas desvantagens são as possíveis complicações inerentes a inserção e manutenção do fio de Kirschner e a necessidade de um segundo procedimento para remoção dos pinos, o que foi realizado no ambulatório da instituição em que este estudo foi realizado.

A técnica de trapeziectomia com tenoartroplastia do músculo palmar longo também busca evitar a migração proximal do primeiro metacarpo, ao utilizar o enxerto tendíneo como um espaçador biológico. Sua principal vantagem é a utilização de enxerto autólogo sem necessidade de implantes sintéticos. Como principais desvantagens dessa técnica, observamos que, apesar de ser do mesmo membro operado, é limitada para pacientes que não têm o músculo palmar longo e as complicações cicatriciais para a retirada do enxerto. Os resultados funcionais positivos notados com o uso desta técnica no presente estudo são concordantes com os da literatura, em que diversos autores 5 15 relatam bons e ótimos resultados.

Ao comparar ambas as técnicas cirúrgicas analisadas, observamos que a trapeziectomia com interposição de tendão do musculo palmar longo não demonstrou superioridade em relação à técnica de Kuhns. Embora não tenhamos encontrado estudos que comparem especificamente essas duas técnicas, os resultados obtidos foram semelhantes a literatura que aborda técnicas cirúrgicas similares. 16 17 18 19 Os achados desta revisão sustentam a eficácia de ambas as abordagens no tratamento da rizartrose. No entanto, com base na Revisão Cochrane, 20 não podemos atualmente fazer recomendações sobre a superioridade de qualquer procedimento cirúrgico sobre outro para essa condição.

Observamos uma correlação positiva entre o TASD e o QuickDASH, resultado consistente com os achados na literatura. 10 21

## Conclusão

As duas técnicas avaliadas se mostraram efetivas para o tratamento de pacientes com rizartrose, mediante avaliação com o questionário TASD, com acompanhamento pós-operatório médio de 30 meses. Não houve superioridade do resultado funcional entre os grupos, comparando as técnicas de trapeziectomia com interposição de tendão ou com distração. Os questionários funcionais TASD específico e o QuickDASH genérico se mostraram equivalentes para mensuração do grau de limitação funcional dos pacientes.
